# Large cholera outbreak in Brong Ahafo Region, Ghana

**DOI:** 10.1186/s13104-017-2728-0

**Published:** 2017-08-10

**Authors:** Charles Lwanga Noora, Kofi Issah, Ernest Kenu, Emmanuel George Bachan, Robert Domo Nuoh, Kofi Mensah Nyarko, Paulina Appiah, Timothy Letsa

**Affiliations:** 1 Ghana Field Epidemiology and Laboratory Training Program, School of Public Health, University of Ghana, Legon, Box LG 13, Accra, Ghana; 2 Regional Health Directorate, Brong Ahafo Region, Ghana Health Service, Box 145, Sunyani, Ghana

**Keywords:** Brong Ahafo region, Atebubu-Amanten, *Vibrio cholera*, Sene west, Nkoranza south

## Abstract

**Background:**

A nationwide outbreak of *Vibrio cholerae* occurred in Ghana in 2014 with Accra, the nation’s capital as the epi-center. The outbreak spread to the Brong Ahafo Region (BAR) which is geographically located in the middle of the country. In this region a review of data collected during the outbreak was carried out and analyzed descriptively to determine the hot spots and make recommendations for effective response to future outbreaks.

**Methods:**

A review of patient records and line lists of cases of cholera reported in all hospitals during the period of the outbreak (July–December 2014) was conducted. Hospitals used IDSR (Integrated Disease Surveillance and Response system) standard case definitions to detect and report cases for management. The GPS coordinates of all districts and health facilities were collected and utilized in the construction of spot maps. We also obtained populations (denominators) from the BAR Health surveillance unit of the Ghana Health Service. All the data thus collected was analyzed descriptively and expressed as frequencies and rates.

**Results:**

A total of 1035 cases were reported, 550 (53.4%) were males and the rest females. Their ages ranged from 1 to 95 years; (mean age of 28.2 ± 19.6 years). The most affected (23.5%) was the 20–29 year old age group. On the 30th July, 2014, a 26 year old male (recorded as the index case of the cholera outbreak in the Brong Ahafo region) with a history of travel from Accra reported to the Nkoranza district hospital with a history of symptoms suggestive of cholera. The reporting of cholera cases reached their peak (17.3%) in week 15 of the outbreak (this lasted 25 weeks). An overall attack rate of 71/100,000 population, and a case fatality rate of 2.4% was recorded in the region. Asutifi South district however recorded a case fatality of 9.1%, the highest amongst all the districts which recorded outbreaks. The majority of the cases reported in the region were from Atebubu-Amanten, Sene West, Pru, and Asunafo North districts with 31.1, 26.0, 18.2 and 9.9% respectively. *Vibrio cholera*e serotype O1 was isolated from rectal swabs/stool samples tested.

**Conclusion:**

*Vibrio cholera*e serotype O1 caused the cholera-outbreak in the Brong Ahafo Region and mainly affected young adult-males. The most affected districts were Atebubu-Amanten, Sene west, Pru (located in the eastern part of the region), and Asunafo North districts (located in the south west of the region). Case Fatality Rate was higher (2.4%) than the WHO recommended rate (<1%). Active district level public health education is recommended on prevention and effective response for future outbreaks of cholera.

## Background

Cholera is an acute diarrheal infection of the intestine caused by ingestion of food or water contaminated with the bacterium *Vibrio cholerae O1* [1, 2]. The disease though can be cured, can largely be prevented through the provision of safe water, good sanitation and enhanced personal hygiene yet in the twentyfirst century the disease remain a threat to lives in many parts of the world particularly in poor, low and middle income countries. Worldwide, an estimated 3–5 million cholera cases and 100,000–120,000 deaths due to cholera are recorded annually [1]. The short incubation period of cholera of a few hours to 5 days, enhances the potentially explosive pattern of outbreaks [1]. Cholera remains a major public health concern in several countries in Africa, Asia and Latin America. Among the WHO regions classified as high cholera burdened countries, there are 69 countries identified as cholera endemic countries with an estimated 1.3 billion people at risk of the disease [3]. There are over 20 million people who are at risk of developing the disease in Ghana. since the re-emergence of cholera in the 1970s from the seventh epidemic Ghana suffered several outbreaks with the most significant one in 1999 in which over 9000 cases and 250 deaths were reported [4]. The disease has remained endemic over the last decade particularly along the coastal communities. Annually, it is estimated that, 41,732 cholera cases occur in the country with and average case fatality rate of 3.8% [3]. Ghana’s cholera cases occur mainly in three regions, Greater Accra Eastern and Central regions of the country [5]. Transmission and spread of cholera has been linked to demographic, socio-cultural, environment and behavioral factors. In 2011, the Government of Ghana and the Netherlands Government and other agencies commenced a developmental project on Water, Sanitation and Hygiene (WASH) to improve on the living conditions of the urban poor in respect of these factors [6]. In addition, UNICEF’s rights-based approach to water, sanitation and hygiene also addresses these challenges through support to the Government of Ghana for both on-ground interventions and the creation of an enabling environment [7] Key WASH interventions, including improving sanitation, provision of safe water, promoting personal hygiene and ensuring food safety in communities and at public gatherings, could effectively control cholera in Ghana.

In compliance with the 2005 International Health Regulations (IHR), the Brong Ahafo Regional Disease Surveillance unit, under Public Health Division of the Ghana Health Service implemented an Integrated Disease Surveillance and Response (IDSR) II system, establishing a network of disease surveillance units across all districts and municipalities that monitors 43 diseases or conditions, including epidemic prone diseases such as cholera [8]. The IDSR is a system with the potential to ensure a reliable supply of information to the national level in order to fulfill IHR requirements. The IHR provide an opportunity to address the threat to international public health security and trade caused by reemerging and emerging infectious diseases including public health emergencies of international concern (PHEIC) [8]. They also provide an excellent opportunity to strengthen surveillance and response systems, and to act as a potent driver for IDSR implementation. Ghana’s IDSR II was launched in 2010 and as part of IDSR II, suspected cholera cases in surveillance units are to be reported and investigated, including laboratory confirmation of etiologic agents [9].

In June 2014, an outbreak of cholera occurred in the Greater Accra Region of Ghana whose capital is Accra. The city has a total population of 4,010,054 with a population density of 1235/km^2^ and there is constant movement of people and goods to and fro the capital to other regions of the country. Subsequently, on 30th of July 2014, a suspected case of cholera was reported at the St Theresa’s Hospital in Nkoranza South District of the Brong Ahafo Region (BAR). Subsequently several cases of acute watery diarrhea were reported from several health facilities across various districts in BAR. Cases were tested with rapid diagnostic test in some districts and later confirmed by culture at the Brong Ahafo (BA) Regional Hospital laboratory for *Vibrio cholerae O1*. Over 1000 cases of cholera and 25 cholera deaths were recorded by the BA regional disease surveillance unit by the end of December 2014 when the outbreak was over. Following these reports a regional team comprising of residents from the Ghana Field Epidemiology and Laboratory Training Program (GFELTP) and the BAR Disease Surveillance Department (DSD) sort to describe the outbreak by Person, Place and Time (PPT) and recommend measures for control of such outbreaks.

## Methods

### Study design and site

**Fig. 1:**
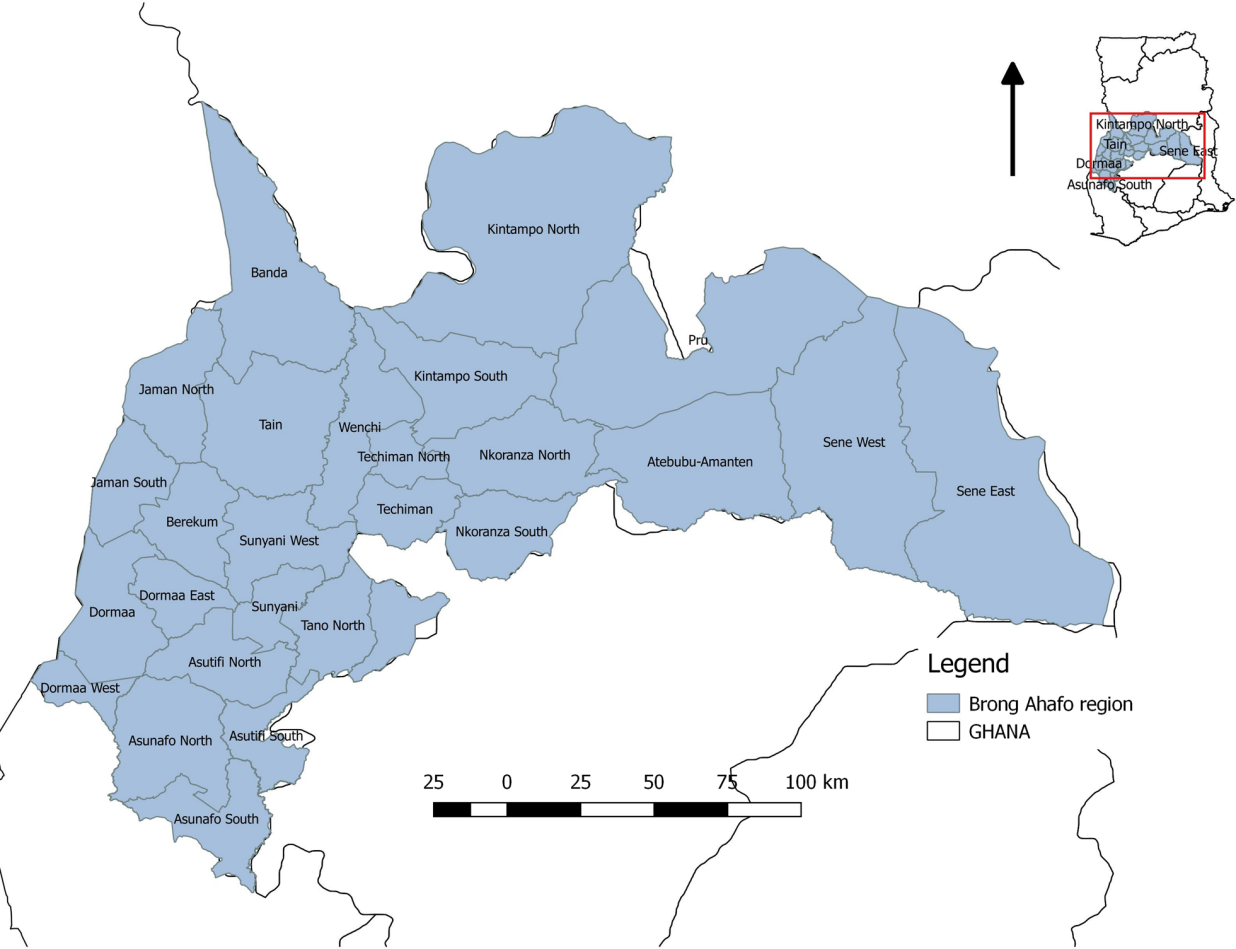
Map of study area, Brong Ahafo Region-Ghana

A descriptive study of cholera cases for 2014 was conducted in all the administrative districts of Brong Ahafo, one of the ten administrative regions of Ghana (Fig. 1). The region with a territorial size of 39,557 km^2^ is the second largest in Ghana and located in the central part of the country with an estimated population of 2.6 million [10]. The region is further divided into 27 administrative districts [8]. The central location of the region and the two main ecological zones in the region; the forest and savanna transition zones makes it vulnerable for the spread of disease of epidemic potential from the northern (e.g. meningitis in the dry season) or southern (e.g. cholera in the rainy season) parts of the country.

Food hygiene, water and sanitation systems in the region is poor with only a relatively low proportion of the population having access to proper sanitary facilities and open defaecation being an increasing phenomenon.

The BAR much like all regions in Ghana runs an integrated disease surveillance system as a strategy to detect and respond effectively to diseases of public health importance, or epidemic potential. There is one regional hospital, 22 district hospitals, 131 health centres, and 467 CHPS (community based health planning and services) facilities conducting facility based disease surveillance. The facility based disease surveillance system in the region is complemented by a community based surveillance system consisting of 4000 volunteers in communities in the region and tasked to report unusual health events on a monthly basis to the sub-district health management teams.

All staff involved in diseases surveillance and the community based surveillance volunteers are provided with periodic training on detecting and reporting 43 priority diseases under surveillance under the IDSR which is the main surveillance system in the region. To ensure that the IDSR system carries out its core functions, trainings are also offered in the use of standard case definitions of diseases under surveillance including cholera.

### Definitions

The IDSR II defines *a suspected cholera case as any patient aged 5* *years or more, with severe dehydration or who dies from acute watery diarrhoea.* However if there is a cholera epidemic, *a suspected case is any person with acute watery diarrhoea, with or without vomiting*. While *a confirmed case is any suspected case in which Vibrio cholerae O1 or O139 has been isolated in the stool* [9].

The WHO recommends the laboratory confirmation of the first cases to ascertain that there is a cholera outbreak. Once the outbreak is confirmed, the clinical case definition is used to detect additional cholera [9] cases and provide treatment [6]. Cases were assessed as having suspected or confirmed cholera based on the IDSR II classification [6]. Only those with *V. cholerae O1 or O139* in the stools were considered as laboratory-confirmed cases.

### Case detection and reporting

The Nkoranza South Municipal Health Directorate was notified of a case of cholera on the 30th July 2014. This was after the case was reported at the Nkoranza district hospital with symptoms suggestive for cholera. The Regional Surveillance unit was notified on the 2nd August, whilst national level got notified on 4th August, 2014.

Standard case definitions for cholera and diarrhoea diseases were used in all health facilities in the region to identify cases. All cases suspected to be cholera were reported immediately under the guidelines of the IDSR system.

### Data collection methods

The research team engaged and sought permission from Regional, Municipal and District Health Disease Surveillance Units and hospital record departments to retrieve records on the outbreak. All Districts and Municipalities are involved in passive surveillance, wherein a minimum set of data is collected and line listed. We reviewed records of cases (line list, OPD and admission folders) from January 2014 to December 2014 in the region. We extracted data on patient demographics, signs and symptoms, laboratory results, and outcome of treatment for analysis. District of the patient’s residence, reporting facility and dates of first presentation were recorded at the District and Municipality levels up to the regional level in separate spreadsheets. District reports were counterchecked with the RDSU database to avoid duplication of cases. We also obtained populations (denominators) from Municipal and District Health Surveillance Units.

### Mapping

All cases obtained from the cholera line list during the period were collected and tabulated according to Districts and Municipalities where the patients came from in Microsoft Access 2010 spreadsheet. Epi info software was used to construct choropleth maps of the sites wherein cholera had been reported to health facilities.

### Data management and analysis

Data was cleaned by running frequencies, checked for consistency and analyzed using Epi-info version 7 and Excel statistical software packages. We analyzed data descriptively and presented as frequencies, relative frequencies, rates, graphs and spot maps.

### Ethical considerations

No ethical approval was required locally for this study, however, permission was sought from the Brong Ahafo Regional Health Directorate and district health management teams and Health facilities in the region to access the data. Findings of this study has since been communicated to all stakeholders in the region.

## Results

### Age and sex distribution of cases

**Fig. 2:**
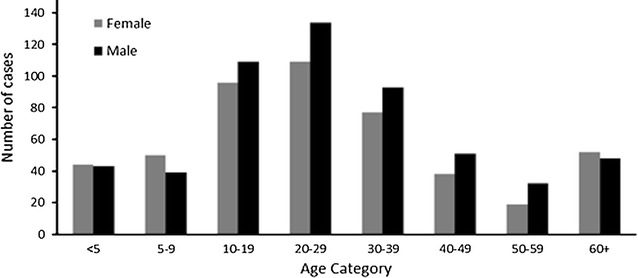
Age category and sex distribution of cholera cases, BAR, 2014

The Brong Ahafo Reginal Health Directorate were alerted of an outbreak of Cholera in the Greater Accra Region of Ghana, However, on 30th of July 2014, a case of cholera was reported at the St Theresa’s Hospital in Nkoranza South District of the Brong Ahafo Region (BAR), eventually an outbreak of cholera occurred with more than 1000 cases reported. The affected population ranged in age from 1 to 95 years with a mean age of 28.2 years and standard deviation of 19.6 years. The age group 20–29 years was reported the highest number of cases with 243/1005 (23.5%). The least (3.5%) affected was the 60–69 years old group (Fig. 2). The highest number of cases was in young male adults, 20–29 year group (Fig. 2). The index case reported was a 26 year old male with a history of travel from Accra (the national capital where an outbreak of cholera had been reported early in 2014). On 30th of July, 2014 he presented to the Nkoranza District hospital (located in the central part of the Brong Ahafo Region) with a history of diarrhoea and vomiting of 24 h duration which started prior to his setting off from Accra. He was admitted at the Nkoranza District Hospital and managed with intravenous infusions, oral rehydration salt (ORS) and tetracycline antibiotics. Rectal sample collected for Laboratory investigation tested positive for *Vibrio cholerae O1* Ogawa sub type. Two days later, two young adult males said to be living in the same vicinity in Accra with the index case also travelled from Accra with similar symptoms (the reason for leaving Accra was to seek health care in Nkoranza where relatives were more likely to settle the bills accruing from their treatment. The two did not have any form of health insurance).

### Distribution of cholera cases by place

There were 1035 cases of cholera recorded in 17 out of the 27 administrative Districts and Municipals of the region. Most of the cases were recorded in Hospitals in the eastern part of the region notably Pru, Atebubu-Amanten, and Sene west districts with 31.1, 26.0, and 18.2% of cases respectively (Fig. 2). The administrative districts which were much more centrally located; Berekum, Kintampo North, Sunyani West, Sunyani Municipal and Nkoranza North cumulatively recorded the lowest proportion of cases (<10%).

**Fig. 3:**
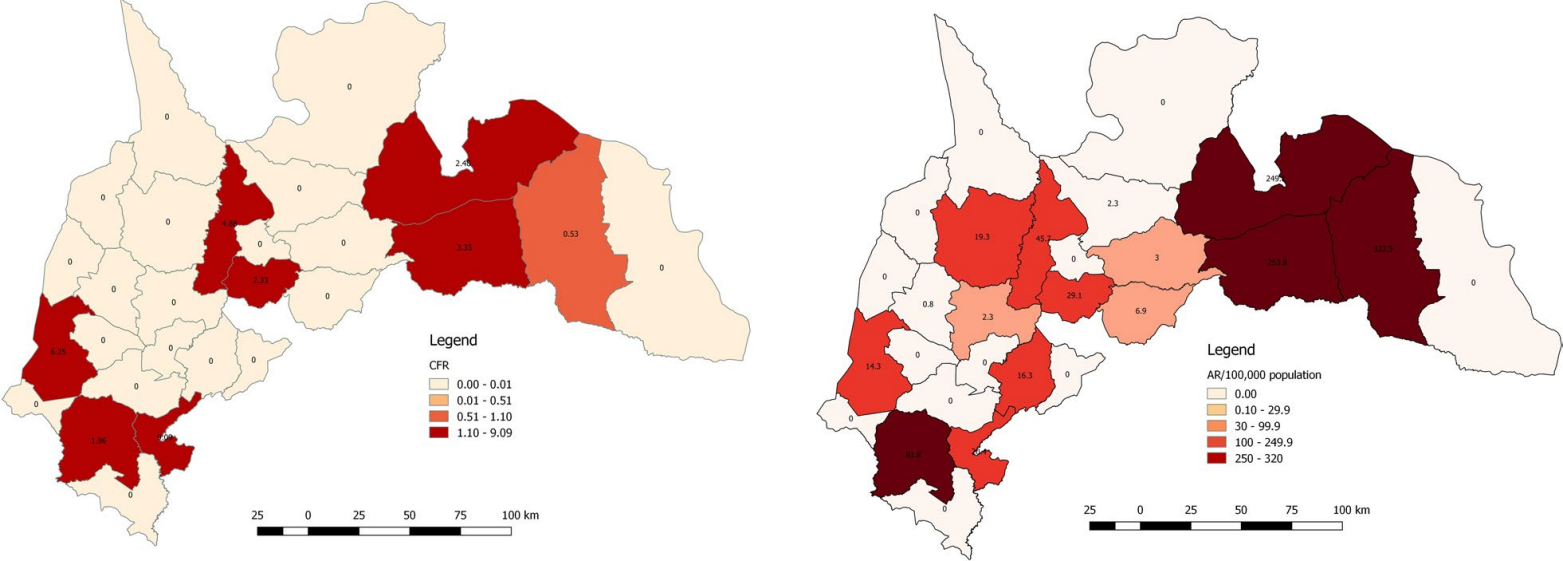
CFR and Hospital presentation (Attack) rate, BAR 2014

All the districts recording fatalities had rates above 2.0%; the highest (9.1%) was in Asutifi South followed by Wenchi 7.7%, Tano South 7.1%, Atebubu-Amanten 3.3%, Pru 2.5%, Techiman 2.5%, Asutifi North 2.0% with only Sene West recording CFR of 0.5% (Fig. 3). Four districts recorded hospital presentation (attack) rates higher than the regional rate of 46/100,000 population; Sene West with an attack rate of 321/100,000 population, Atebubu-Amanten 254/100,000 population, Pru district 249/100,000 population and Asunafo North 82/100,000 population (Fig. 2). Berekum and Kintampo North districts recorded the lower ARs with 0.8 and 1.0 respectively (Table 1). There were no cases recorded in 11 of the 27 districts in the region (Fig. 3).

**Table 1:** Cases of cholera reported in health facilities by district, BAR, 2014

Municipal/district	Cases	Population	AR/10,000	Deaths	CFR	%
Asutifi south	11	52,844	20.8	1	9.1	1.1
Dormaa municipal	16	112,111	14.3	1	6.3	1.5
Wenchi municipal	41	89,739	45.7	2	4.9	4.0
Atebubu-Amanten	269	105,938	253.9	9	3.3	26.0
Pru	322	129,248	249.1	8	2.5	31.1
Techiman municipal	43	147,788	29.1	1	2.3	4.2
Asunafo north	102	124,685	81.8	2	2.0	9.9
Sene west	188	58,299	322.5	1	0.5	18.2
Tain	17	88,104	19.3	0	0.0	1.6
Tano north	13	79,973	16.3	0	0.0	1.3
Berekum municipal	1	129,628	0.8	0	0.0	0.1
Kintampo north	1	95,480	1.0	0	0.0	0.1
Sunyani west	2	85,272	2.3	0	0.0	0.2
Nkoranza south	7	100,929	6.9	0	0.0	0.7
Nkoranza north	2	65,895	3.0	0	0.0	0.2
Total	1035			25	2.4	100

### Laboratory results

Out of the 199 suspected cases investigated by culture at the BA Regional Hospital Laboratory, Sunyani, 153 (76.9%) were confirmed positive for *Vibrio cholera*, and Ogawa serotype from rectal swabs and stool samples. Of the 153 cases that were confirmed; Asunafo North 97, Techiman 26, Wenchi 9, 5 each from Atebubu-Amanten, Dormaa East, and Nkoranza South. The rest were Sunyani Municipal 3, Pru district 3 confirmed cases.

**Fig. 4:**
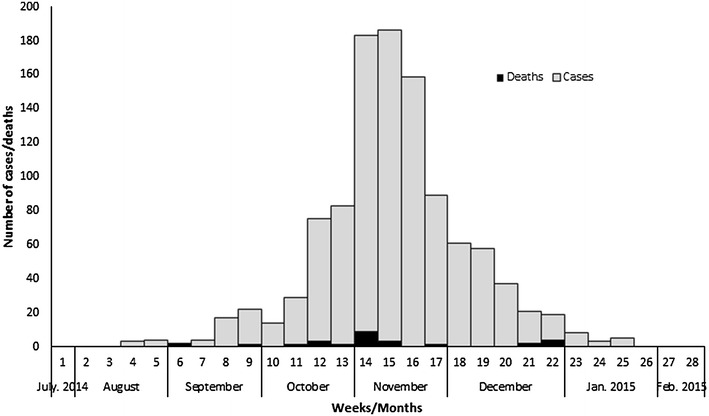
Cholera cases by date of onset, BAR, 2014

Time trends of cholera cases suggested that on the average, 45 cholera cases occurred in the region each week from the beginning of August 2014. The epi curve of cases demonstrates that the outbreak start around 30th July 2014 with just one case (a 26 year old man from Nkoranza South identified as the index case), Cases steadily rose and peaked in week 15 of the outbreak with 17.3% of cases and gradually reduced to 5% in week 25 before closing into 0% in the subsequent 3 weeks (Fig. 4).

## Discussion

The detection of a little over 1000 cases of cholera and a case fatality of 2.5% pointed to a major outbreak in the Brong Ahafo Region of Ghana. A combination of factors; the rainy season of the year, contaminated sources of drinking water, poor food hygiene and sanitation practices probably contributed to the large number of cases in the three districts located in the eastern part of the region. This study is limited in not exploring further the factors that accounted for an outbreak of such magnitude and why it was not prevented despite alerts earlier in the year of cases in the southern parts of the country [11]. It also cannot be ascertained whether the index case in the region is the source of the outbreak or there were simultaneous occurrence of cases from other sources. Despite these limitations this study provides a snapshot of the scale of the outbreak for the strengthening of the surveillance system to respond to future outbreaks. It also presents an opportunity for clinicians to learn from this experience to improve their cholera case management skills in the future.

In documenting which age groups were affected it is clear that the 20–29 year group has the highest numbers of cases similar to other findings [5, 11, 12]. This is an age group in which the males are more likely to eat food sold by vendors at lorry parks, markets and other public places were the level of food hygiene cannot be guaranteed. It is not surprising therefore the history collected from the index case and two other cases pointed to the fact that they probably would have contracted the disease through food sold by vendors at a very unhygienic suburb of Accra.

Three neighbouring districts (Atebubu-Amanten, Pru, and Sene West) with a history of a recent outbreak in 2012 recorded the highest number of cases [13]. It is possible that these districts are gradually becoming endemic for cholera unless sources of infection such as their seasonal contamination of water sources and open defecation are controlled by the local government authorities and other stakeholders. In spite of its lying in the South western part of the region (where sanitation and hygiene is much improved) the Asunafo North shares similar characteristics with the three earlier mentioned districts hence the high number of cases recorded.

Despite having large numbers of cases the case fatality in these districts were low, as it is possible the experience of the 2012 epidemic gave the population a high index of suspicion and the tendency to report cases early for management at health facilities. It is however worrying that these same districts could not send enough samples to the Regional hospital laboratory for culture and confirmation. Secondly the study fails to capture the level of use of rapid diagnostic tests (RDTs) to classify all the reported cases or ascertain the degree of sensitivity or specificity of the kits utilized. It means most of the cases could have been classified as cholera on the basis of epidemiological linkages. On the other hand, culture for cholera samples in the whole region is limited to only the Regional hospital laboratory thus reducing access to improving laboratory confirmation for the cholera outbreak. The higher CFR recorded in Asutifi South, Dormaa, Wenchi, Atebubu, Amantia, Pru, Techiman and Asunafo North districts were possible due to the several reasons resulting in late reporting, diagnosis and treatment. Many of these districts are farming communities where natives settle very far away from the capital where the district hospitals are located. Records reviewed showed many of these patients were referred from a lower health facility and in some cases, the facilities had no staff or hospitals to operate hence the referral.

In subjecting the surveillance system to scrutiny, it needs further investigation as to how the Sene East district with similar or poorer environmental conditions to the Sene West, Pru and Atebubu districts. The lack of a district hospital in the Sene East district possibly means all their cases were reported and attributed to the district hospitals in Pru, Atebubu, and Sene West thus not presenting a true picture of the places of residence of some of the cases during this outbreak.

Each rainy season in the tropics is normally accompanied by an increase in diarrhoeal diseases including cholera when the environmental and socio-cultural conditions are ripe for an outbreak [10]. Open defecation and polluted water sources with the onset of the rains results in possibly cholera contaminated water and food being consumed by people (mostly young adult males) who purchase and consume food and water from public vendors. The peak in the numbers of cases reported in November (when the rainy season is almost ended) needs further review and analysis to provide an explanation for the trend.

The core functions of case detection, documentation and reporting has not been very effective as there were delays in reporting the index case from lower levels of the health surveillance system to the regional and national levels. The support functions of the surveillance system to include communication, logistics, stakeholder collaboration also might not been well spelt out to combat this and any future outbreaks of cholera in the region.

## Conclusion

An outbreak of cholera due to *Vibrio cholerae O1* occurred in the Brong Ahafo region of Ghana in 2014, the second in as many years (the last in 2012) raising questions to decreasing intervals of cholera epidemics in the region. The overall CFR was higher than the WHO acceptable rate of <1%. The high number of cases amongst young adult males who probably consume food and water prepared and sold under unhygienic conditions from vendors calls for increased health education on food hygiene. The outbreak is described as large in magnitude occurring in more than 80% of districts in the region given that the region is known as the cleanest in Ghana. Three districts in the eastern part of the region are becoming endemic for cholera raising concerns for all stakeholders to collaborate more closely to reduce open defecation, and polluting of water sources especially during the rainy season of each year.

Core and support functions of the disease surveillance system with focus on cholera have to be strengthened with planning, public education effective mobilization and utilization of logistics in the management of cases being given prominence.

## Abbreviations

BAR: Brong Ahafo Region
DSD: Disease Surveillance Department
GFELTP: Ghana Field Epidemiology and Laboratory Training Programme
IDSR: Integrated Disease Surveillance and Response
IHR: International Health Regulations
OPD: outpatients department
ORS: oral rehydration salt
PHEIC: public health emergencies of international concern
PPT: person place and time
RDSU: Regional Disease Surveillance Unite
UNICEF: United Nations Children’s Fund
WASH: water sanitation and hygiene
WHO: World Health Organization
